# Working together: a multi-component intranasal vaccine provides synergistic protection against COVID-19

**DOI:** 10.1038/s41392-025-02508-0

**Published:** 2025-12-22

**Authors:** Jessica A. Breznik, Chris P. Verschoor

**Affiliations:** 1 Department of Molecular and Cellular Biology, College of Biological Science, University of Guelph, Guelph, ON Canada; 2 Department of Medicine, McMaster University, Hamilton, ON Canada; 3 Health Sciences North Research Institute, Sudbury, ON Canada; 4 NOSM University, Sudbury, ON Canada

**Keywords:** Molecular medicine, Immunology

**Fig. 1:**
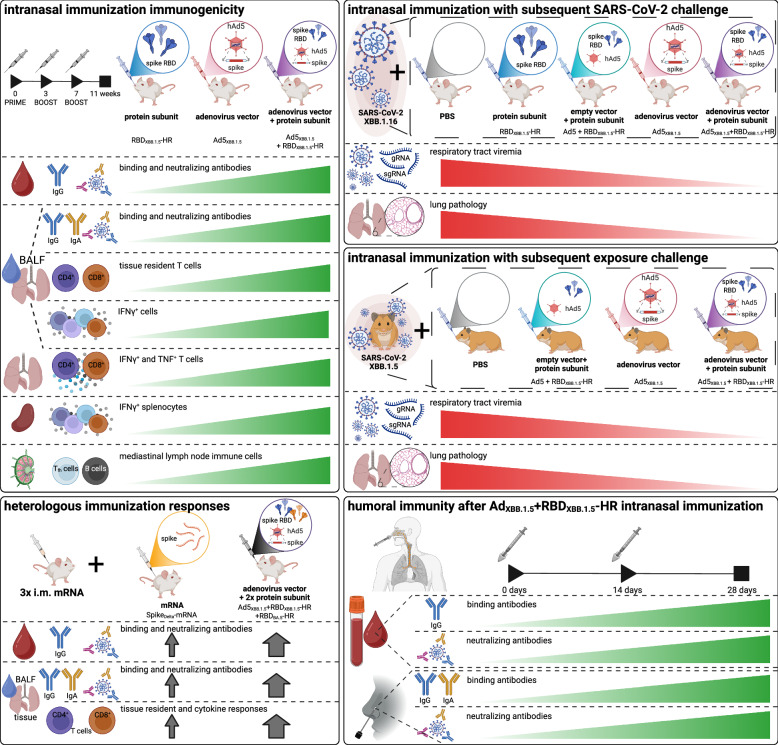
A summary of the major experiments used to support the efficacy of the multi-component intranasal COVID-19 vaccine by Hong and colleagues

In their recent publication in *Nature Biomedical Engineering*, Hong and colleagues^1^ report on the development of COVID-19 intranasal vaccines that combine adenoviral vector and protein subunit vaccine platforms. A two-component vaccine comprised of a human adenovirus expressing the full spike protein and a recombinant spike protein receptor binding domain generated mucosal and systemic immunity against live viral challenge while preventing transmission in animal models, and was shown to be well-tolerated, safe, and effective at inducing antibody responses in humans (Fig. 1).

The emergency response to the COVID-19 pandemic has led to the development of a number of different SARS-CoV-2 vaccines. The most commonly administered vaccines have been mRNA or inactivated virus-based platforms, partly due to their relatively low cost and ease of development,^2^ followed by viral vector and recombinant protein (i.e. subunit) based approaches. Viral vector vaccines employ viruses, such as human adenovirus serotype 5 (Ad5), which are modified to express immunogenic components of the pathogen of interest. This results in the transient expression of the target protein in host cells, while inducing an anti-viral response through pathogen-recognition receptors such as STING and toll-like receptor (TLR)-9. Although the anti-viral response is beneficial, acting like a ‘natural’ adjuvant to boost overall immunogenicity, immunity arising from prior exposure to viruses of the same family as the vaccine vector has been shown to limit effectiveness of these platforms.^2^ Recombinant protein platforms employ synthetic versions of the target protein and are therefore advantageous when aiming to target specific components of the pathogen. Although recombinant proteins are known to exhibit very good safety profiles, they also tend to be poorly immunogenic. Thus, these vaccines often require supplementation with an adjuvant, such as purified saponin (i.e. Matrix-M) and cytosine phosphoguanine (CpG), which tend to increase the risk of acute adverse events.^2^

Although each vaccine platform targeting SARS-CoV-2 has a unique mixture of benefits and disadvantages, the vast majority employ an intramuscular route of administration, which is very safe and can be easily delivered in a variety of clinical settings. However, many immunologists have challenged this approach since it often fails to mimic the natural route of infection, and is therefore limited in conferring a robust protective immune profile where the host is most likely to be exposed.^3^ As such, the induction of secretory IgA and activation of local resident memory and effector cells (i.e. mucosal immunity) is presumed to be superior to serum IgG and circulating memory and effector cell responses (i.e. systemic immunity) in preventing infection by respiratory pathogens, although a combination of both would likely result in optimal host protection. Mucosal vaccines, which are administered intranasally or through inhalation, have shown promise to generate strong mucosal immune responses; however, specific challenges exist when attempting to adapt intramuscular platforms for mucosal delivery, resulting in reduced vaccine effectiveness.^3^

In their recent publication in *Nature Biomedical Engineering*,^1^ Weiqi Hong and colleagues used a novel approach to overcome many of these challenges, developing a two-component COVID-19 intranasal vaccine comprised of a human Ad5 vector expressing the full spike protein of the Omicron XBB.1.5 variant (Ad5_XBB.1.5_) and a recombinant spike receptor binding domain (RBD) protein (RBD_XBB.1.5-_HR). The viral vector facilitates effective transport across the mucosal barrier, since adenoviruses normally infect via this route, while acting as an adjuvant to boost the uptake and presentation of the recombinant protein. To demonstrate the flexibility of their approach, the authors also examined the effectiveness of multi-component vaccines targeting the Omicron BA.5 variant (i.e. Ad5_BA.5_ and RBD_BA.5_-HR) and both the Omicron XBB.1.5 and BA.5 variants (i.e. Ad5_XBB.1.5_, RBD_XBB.1.5_-HR and RBD_BA.5_-HR).

Murine studies confirmed that Ad5_XBB.1.5_ + RBD_XBB.1.5_-HR vaccine immunisation responses were present systemically (i.e. blood, spleen and/or lymph nodes) and within respiratory mucosal tissues (i.e. nasal and bronchoalveolar lavage and/or lung tissue), through measurements of anti-RBD IgG and IgA titres, pseudovirus and live virus neutralising antibody titres, and spike-specific germinal centre B cells, as well as CD4^+^ and CD8^+^ memory T cells, T follicular helper cells, and T cell interferon (IFN)-ɣ and tumour necrosis factor (TNF) production after spike protein stimulation. This was similarly shown using a three-component Ad5_XBB.1.5_ + RBD_XBB.1.5_-HR + RBD_BA.5_-HR vaccine, demonstrating the flexibility of this multivalent platform to incorporate additional antigens and potentially broaden protection against other SARS-CoV-2 variants. Importantly, the authors show that heterologous vaccination with three doses of an intramuscular mRNA vaccine, followed by their three-component platform, enhanced systemic and respiratory mucosal immunity beyond that conferred by intramuscular vaccination alone. Comparisons of immunisation responses to Ad5_BA.5_, RBD_BA.5_-HR, and/or Ad5_BA.5_ + RBD_BA.5_-HR in a series of genetic knockout mice, and examination of lung tissue using single-cell RNA-sequencing, identified that the adenoviral vector had a STING-dependent effect that enhanced dendritic cell phagocytosis, antigen processing, and presentation of the RBD_XBB.1.5_-HR protein subunit. Intriguingly, findings suggested that even an empty adenoviral vector could be used as a vaccine adjuvant.

Effectiveness of the two-component vaccine against SARS-CoV-2 was evaluated by post-vaccination live infection challenge in mice and post-vaccination contact/air exposure in hamsters. Evaluation of genomic and subgenomic RNA in throat swabs, nasal turbinates, trachea, and lung tissues, and lung tissue pathology, indicated that the Ad5_XBB.1.5_ + RBD_XBB.1.5_-HR vaccine provided robust protection against infection. To evaluate safety and immunogenicity in humans, the authors administered either a low or high dose of their intranasal Ad5_XBB.15_ + RBD_XBB.1.5_-HR vaccine, with a 14-day interval prime-boost, to 70 adults who had not received a COVID-19 vaccine or experienced an infection in the previous 3 or 6 months, respectively. The vaccine was well-tolerated with only self-limiting mild adverse reactions, and produced robust mucosal and systemic humoral immune responses as indicated by the investigation of serum and nasal swab samples. However, it is worth noting the limitations of this set of analyses. Namely, the lack sample collection beyond 28 days post-vaccination and the omission of any assays evaluating cellular responses.

At the time of writing, five mucosal SARS-CoV-2 vaccines have received geographically limited approval for human use.^3^ These vaccines employ different platforms (i.e. protein subunit, adenoviral vector, or live attenuated influenza virus), and incorporate components of the ancestral SARS-CoV-2 spike protein. Continued focus on full length and/or RBD regions of spike proteins within respiratory mucosal vaccines, as in the work by Hong and colleagues, may not provide long-term protective immunity against future variants, as mutations within the SARS-CoV-2 spike protein have significantly contributed to immune escape. Non-spike proteins, including envelope, membrane and nucleocapsid, are commonly targeted by antigen-specific T-cells generated following infection and have been shown to correlate with protection in individuals where exposure was likely.^4^ Further, vaccines coding both spike and nucleocapsid antigens have been demonstrated to provide long-term protection in mouse models of infection.^5^ Hence, the development of multi-antigenic COVID-19 mucosal vaccines that contain spike and non-spike proteins and target mucosal immunity could present a next-generation solution that offers broad and durable immunity against current and future variants of SARS-CoV-2. Taken together, the work by Hong and colleagues compellingly demonstrates that integration of adenoviral vectors as adjuvants of other vaccine platforms, or within combined platform vaccines, may provide novel strategies to induce robust mucosal immunity and may be of importance in the future vaccine development.
